# Outcome of four years experience in laparoscopic ventral hernia repair

**DOI:** 10.12669/pjms.314.6326

**Published:** 2015

**Authors:** Dileep Kumar, Hina Khan, Muhammad Shamim Qureshi

**Affiliations:** 1 Dileep Kumar, Registrar General Surgery, Jinnah Postgraduate Medical Centre (JPMC), Karachi, Pakistan; 2 Hina Khan, Postgraduate General Surgery, Jinnah Postgraduate Medical Centre (JPMC), Karachi, Pakistan; 3 Muhammad Shamim Qureshi, Associate Professor of General Surgery, Ward 2, Jinnah Postgraduate Medical Centre (JPMC), Karachi, Pakistan

**Keywords:** Ventral hernia, Seroma, Postoperative Ileus, Laparoscopic ventral hernia repair (LVHR)

## Abstract

**Objective:**

To find out the short term outcomes of laparoscopic ventral hernia repair (LVHR) during the last four years.

**Methods:**

It was a descriptive and prospective case series of 53 consecutive patients out of 107 at Department of General Surgery, Jinnah Post Graduate Medical Center, Unit II, Karachi, from January 2009 to December 2012. These patients were admitted through out patient department with complain of lump, pain and discomfort. Most of the patients were obese. All patients were clinically examined and baseline investigations done. Fifty three (49.5%) patients underwent laparoscopic repair with mesh placement and remaining 54 by open surgical repair.

**Results:**

Among 53 patients, mean age was 46 years range (30 - 55). While females were 33(62.2%) and males 20(37.7%). We observed variety of hernias, in which midline and epigastric hernia were predominant. The commonest symptom was lump and dragging sensation. The duration of symptoms ranged between 6 months to one year. About 53 patients (49.5%) had laparoscopic repair with mesh placement. Average hospital stay was two days. Out of 53 patients, 4 (7.5%) had cellulitis at trocar site, seroma in 2(3.7%), 2(3.7%) patient complained of persistent pain postoperatively, port site minor infection was in 2(3.7%) patients, while conversion to open approach was done in 2 (3.7%), postoperative ileus was observed in one (1.8%) patients.

**Conclusions:**

This study provides the evidence that, laparoscopic repair with mesh placement in ventral hernia is safe and effective approach compared to open surgical procedure. It has a low complication rate, less hospital stay and low recurrence.

## INTRODUCTION

Ventral hernia is defined as Primary anterior abdominal wall and Incision hernia not including the groin.^1^ About two million laparotomies are performed in the United States each year leading to an incisional hernia rate of 3% to 20%,^2^ requiring repair of 90,000 ventral hernias annually. There are number of risk factors that lead to hernia to occur; like wound infection, morbid obesity, immunosuppression, previous operations, prostatism, and surgery for aneurysmal disease^3^. Hernia defect can form within first 5 years of surgery, but can occur late as well.^3^

Up till now, many procedures have been described for ventral hernia repair. Primary repair with suture approximation requires laparotomy, with the recurrence rate of 41% to 52% during long-term follow-up.^4^ In open repair, wide area of dissection is required, which contributes to an increased incidence of wound-related complications (12% or higher).^5^,^6^ Therefore surgical treatment of ventral hernias has changed dramatically over the past decades by the introduction of laparoscopy and prosthetic biomaterials for reinforcement of the abdominal wall.

LVHR was first done by Karl LeBlanc in 1992.^1^ He performed Intraperitoneal onlay mesh repair (IPOM) reported short hospital stay, 0 – 9% recurrence and less complications. The basic technique for repair is access to the abdominal cavity, adhesiolysis and repair of defect. There are still many controversies regarding the type of mesh and fixation of mesh. An ideal mesh should be strong, pliable, non-allergenic, non-biodegradable, non-carcinogenic and should stimulate adequate fibroblastic activity. Prosthetic material can be polypropylene, polyester and ePTFE. The first two meshes are ideal for use where they do not come in contact with the abdominal viscera, like laparoscopic repairs of inguinal hernias - TAPP or TEP. Though some surgeons use it as intra-abdominal placement for repair of ventral and incisional hernias, this is not advisable since literature reports of complications of bowel adhesions, bowel obstruction, fistulization and erosion into abdominal viscera even after many years.^7^ Although complications are less common with laparoscopic repair, but wound and mesh related complications, persistent postoperative pain, bowel obstruction, postoperative ileus and rarely cardiac tamponade^8^ can occur. Our objective ws to find out the short term outcomes of laparoscopic ventral hernia repair (LVHR) during the last four years

## METHODS

This study comprises of total 107 patients, out of which 53(49.5%) were scheduled for laparoscopic ventral hernia repair, while other 54 underwent open repair. These patients were admitted through outpatient department, from January 2009 to January 2013 with random sampling. Most of the patients complained of lump, pain and discomfort. Most of the patients complained of lump, pain and discomfort. Majority were obese. All patients were clinically examined and baseline investigations done. Fifty three patients underwent laparoscopic repair with mesh placement and remaining fifty four by open surgical repair. Inclusion criteia for laparoscopic repair was patients who were obese, having hernial defect size of >4 cm, history of multiple abdominal surgeries and American Society of Anesthesiologist (ASA) I, II, while high risk patients (ASA III, IV, patients having CLD / coagulopathy) were excluded. Three different types of meshes were used according to availability and affordability. Number of complications were observed on follow up. The data of different variables like age, gender, size of defect, postoperative hospital stay and complications were collected retrospectively and assessed by SPSS version 10. The study was approved by the JPMC Ethics Committee on 27 February 2014.

## RESULTS

There were total 53 patient who had laparoscopic ventral hernia repair, out of which females were 33(62.2%). Mean age was 46.6 years. There were variety of ventral hernia, in which majority were mid line incisional hernias 18(33.9%) and epigastric 10(18.8%). Fig-I.

**Fig.1:**
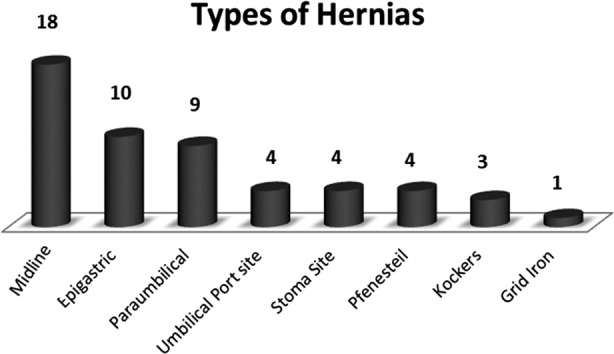
Types of Hernias.

We have been shifting our open approach towards laparoscopic repair from 2009, and per year cases are shown in Table-I. Our 32 cases were of ASA – I and 21 of ASA – II. Operating time was ranging from 50 – 150 minutes. We used Prolene mesh (15 × 15 and 30 × 30) with omental covering in 47(88.6%) patients, Dual mesh, 15 × 15 in 4(7.5%) and Physio Mesh in 2(3.7%).

**Table-I:** Number of cases (2009-2014)

Year	Number of cases
2009	4 (7.5%)
2010	10(18.8%)
2011	12(22.6%)
2012	26(49.05%)
2013 (January)	1(1.8%)

Postoperative recovery was uneventful in all. Patients were followed for the complications and we found cellulitis in 4(7.5%) pain at trocar site, 2(3.7%) patients had seroma, 2(3.7%) prolonged pain, conversion to open in 2(3.7%) secondary injury to large bowel, while 2(3.7%) patients developed wound infection and 1(1.8%) prolonged ileus, while there was no hematoma. The hospital stay rages from 3 – 7 days. Fig-II.

**Fig.2:**
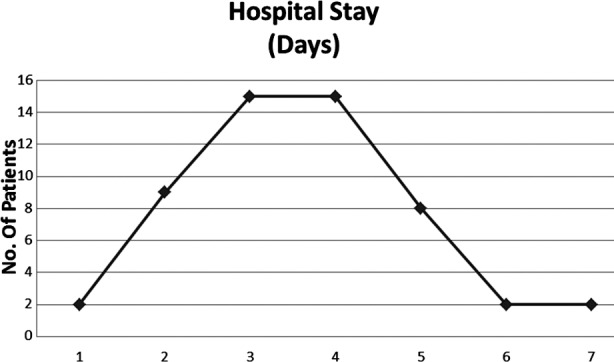
Hospital Stay.

## DISCUSSION

Incisional hernia is the most common long term complication of abdominal surgery and an important source of morbidity. Treatment involves further major surgeries. Several large studies on laparoscopic ventral hernia repair have been reported. This technique has proven to be a safe and feasible alternative to open mesh repair.^9^,^10^ The main advantage of this minimally invasive approach is a decrease in the rate of major wound complications and early return to work.^11^

In early 1960s, Before the introduction of polypropylene mesh, incisional hernias were repaired by direct suture techniques,^3^ which included simple fascial closure, with recurrence rate of 7 - 44%.^3^,^12^

Types of mesh is now a days debatable issue due to diffeences between the surgeons. The choice of mesh may therefore be difficult in clinical practice. Usher^14^ introduced knitted monofilament polypropylene mesh in 1963. Micheal.^15^ reported the use of prolene mesh with omental covering, supported prolene as it is porous, cost effective, easily available, has good intra corporeal handling and causes rapid fibrinous fixation to musculofascial layer. We also use the same technique of omental covering over prolene mesh in 47(88.6%) patients. “Naked” Polypropylene mesh in the abdominal cavity is no longer acceptable owing to the long term risk of adhesion formation.^15^ Non absorbable or composite mesh is recommended for hernia repair where it does not come in contact with the bowel. Dual mesh is used in two choices: one is a solid sheet and the other is perforated to allow for greater tissue incorporation. There are various other types of mesh available, but we used dual mesh in 4(7.5%) patients and physio – mesh in only 2 (3.7%) due to cost.

Many studies have reported better surgical outcome with two dual sided ePTFE mesh,^1^,^16^ we mostly used polypropylene due to economic consideration. Adhesions seems to be more frequent with polypropylene mesh but it does not seem to affect the repair success or recurrence rate.^17^,^18^ Now a days intestinal obstruction and enterocutaneous fistula formation now seems to be subsiding, although debate persists.^12^,^17^-^19^ Vrijland et al.^19^ observed rare rate of enterocutaneous fistula after incisional hernia repair with intraperitoneal placement of polypropylene mesh. Franklin et al.^18^ found no such complications in series of 384 patients with ventral hernia, when used polypropylene mesh in 75% of cases. We also didn’t notice these complications.

Bowel injury during adhesiolysis is a commonest fear in laparoscopic incisional hernia repair procedure.^16^,^17^ We had single large bowel injury as complication, due to which we converted to open approach. Seroma, wound infection including trocar site infection, ileus, haematoma and pain are common postoperative complications of laparoscopic repair. Our 4 (7.5%) patients developed cellulitis at trocar site, prolonged pain in 2(3.7%) and wound infection in one patient only. In one of the Indian study,^20^ seroma was 18%, while we had 3.7%, which resolved spontaneously. Our single patient developed ileus, which corresponded to the other author.^20^ The recurrence rates with laparoscopic incisional hernia repair vary between 0 -10%.^17^,^21^ We have not observed any recurrence in our patients. We conclude that laparoscopic repair is an appropriate approach for ventral hernia repair as it results in good repair and low wound complications and recurrence.

### Limitations of the study

Small sample size and short term follow up as this a relatively newer techniques.Long term follow up will confirm the relevance of this technique.
